# “Gut–brain axis”: Review of the role of the probiotics in anxiety and depressive disorders

**DOI:** 10.1002/brb3.1803

**Published:** 2020-09-10

**Authors:** Eleonora Gambaro, Carla Gramaglia, Giulia Baldon, Emilio Chirico, Maria Martelli, Alessia Renolfi, Patrizia Zeppegno

**Affiliations:** ^1^ Department of Translational Medicine Institute of Psychiatry Università del Piemonte Orientale Novara Italy; ^2^ Psychiatry Ward Maggiore della Carità University Hospital Novara Italy

**Keywords:** anxiety, depression, gut–brain axis, inflammation, probiotics

## Abstract

**Background:**

Depressive disorders are the leading cause of disability worldwide and together with anxiety contribute to a very high burden of disease. Therefore, improving their treatment is a significant medical research target: The role of probiotics is a topic of great interest for the current research in this field.

**Objectives:**

To explore the current literature about the impact of probiotics on anxious and depressive symptoms.

**Methods:**

Scoping review following the PRISMA guidelines.

**Results:**

The selection process yielded 23 studies. Probiotics positively affected depressive symptomatology and anxiety symptoms according to 53.83% and 43.75% of the selected studies, respectively. Among the studies assessing inflammatory biomarkers, 58.31% found they were decreased after administration of probiotics.

**Conclusion:**

The results emerging from the existing literature about probiotic supplementation for depression treatment are encouraging, but further research is needed considering the shortage of clinical trials on this topic and the heterogeneity of the samples analyzed.

## Graphical abstract

(a). Recently, the concept of “gut‐brain axis” has been of great interest for the research and the hypothesis that probiotic treatment could improve the symptomatology and the inflammatory status of the patients affected by depression has emerged. (b). Many trials, studying the effects of probiotic intake on depressive symptoms and inflammatory biomarkers status, were published and their results looks promising, even if only few studies included a population with a diagnosis of depression. 3. For this reason, other trials and reviews are needed to increase the knowledge in this branch of research.

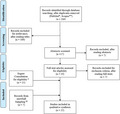

**Abbreviations**
BAI: Beck Anxiety Inventory
BDI: Beck Depression Inventory
CFU: Colony‐forming Unit
CNS: Central Nervous System
CRP: C‐reactive Protein
DASS: Depression, Anxiety and Stress Scale
EQ‐5D‐5L: EuroQoL Dimensions and 5 Levels Measure of Health and Wellbeing
FOS: Fructooligosaccharides
GAF: Global Assessment of Functioning
GSH: Glutathione
HADS‐A: Hospital Anxiety and Depression Scale—Anxiety
HADS‐D: Hospital Anxiety and Depression Scale—Depression
HAQ: Health Assessment Questionnaire
HCs: Healthy Controls
HOMA‐IR: Homeostatis Model Assessment of Insulin Resistance
HRQoL: Health‐related Quality of Life
HSCL‐90: Hopkins Symptoms Checklist—90
IBS: Inflammatory Bowel Syndrome
IBS‐QoL: Irritable Bowel Syndrome—Quality of Life
LEIDS‐R: Leiden Index of Depression Sensitivity—R
MADRS: Montgomery–Asberg Depression Rating Scale
MDD: Major Depressive Disorder
MINI: Mini International Neuropsychiatric Interview
NASH: Nonalcoholic Steatosis Hepatitis
NOS: Newcastle Ottawa Scale
NS: Not Specified
NSAID: Nonsteroidal Anti‐inflammatory Drugs
OTC: Over The Counter
POMS: Profile of Mood States
PPI: Proton‐pump Inhibitors
PRISMA‐ScR: Preferred Reporting Items extension for scoping reviews
QIDS‐SR16: Quick Inventory of Depressive Symptomatology, 16 Items, Self‐Report
RCTs: Randomized Controlled Trials
SDs: Standard Deviations
SF‐36: Short Form Health Survey—36
SPAI‐23: Social Phobia and Anxiety Inventory—23
STAI‐Y: State Trait Anxiety Inventory—Y
TNF: Tumor Necrosis Factor
VSI: Visceral Sensitivity Index

## 1 INTRODUCTION

According to the latest data published by the WHO (Estimates, 2017), depression has become one of the main topics in medical research. Depressive disorders are the leading cause of disability worldwide, with a huge cost for healthcare institutions. More than 300,000,000 people are affected by depression, corresponding to approximately 4.4% of the world population. (Estimates, 2017).

While several effective pharmacological and nonpharmacological treatments for depression are available, many studies have shown that only about 46% of treated patients undergo symptomatic remission after combined treatments. (de Maat, Dekker, Schoevers, & de Jonghe, 2007).

Anxiety disorders represent a considerable health problem worldwide as well (Kessler, Petukhova, Sampson, Zaslavsky, & Wittchen, 2012), involving different interacting factors such as genetic, neurobiological, and socio‐psychological ones. (Bandelow et al., 2016).

Anxiety and depression are frequently comorbid in the population, reaching a prevalence of 25% worldwide. About 85% of depressed patients have concurrent anxiety symptoms, and, similarly, patients with a diagnosed anxiety disorder show comorbid depressive symptoms in about 90% of cases. (Tiller, 2013) Despite many effective drugs are available for treating these disorders, up to 40% of patients do not take any medication, and even in those under medication, complete remission of symptoms is achieved in about half of cases. (Tiller, 2013) For these reasons, further research is required to identify effective treatment, improve adherence to therapy, and achieve recovery from depressive and anxious disorders.

In recent years, several experimental works have investigated the effect of probiotics in the treatment of neuropsychiatric disorders. (Burokas, Moloney, Dinan, & Cryan, 2015).

The gut is colonized by 10^13^–10^14^ microorganisms (Burokas et al., 2015), known as gastrointestinal microbiota, which plays a role in human health (Guarner & Malagelada, 2003; O’Hara & Shanahan, 2006), and contributes to the development of different diseases. Several authors focused their attention on the interaction between the gut microbiota and the central nervous system, via endocrine, neural, and immune pathways, with effects on brain function, cognition, and behavior. (Mayer, 2011) The term gut–brain axis has therefore been proposed (Burokas et al., 2015; Collins, Denou, Verdu, & Bercik, 2009) to refer to the bidirectional communication between the gastrointestinal tract and the central nervous system. (Wang & Kasper, 2014).

Besides the possible role of the gut–brain axis in the pathogenesis of depression, several studies have investigated the cytokine hypothesis of depression (Leonard, 2018; Miller & Raison, 2016), according to the finding of increased levels of pro‐inflammatory cytokines in depressed patients (Duivis, Vogelzangs, Kupper, de Jonge, & Penninx, 2013; Lamers et al., 2019), and of possible improvements in depressive symptoms after anti‐inflammatory treatments. A recent review showed that low‐dose aspirin treatment is not only safe and well‐tolerated but also potentially efficacious for “improving depressive symptoms in both unipolar and bipolar depression” (Ng et al., 2019). Furthermore, pro‐inflammatory stimuli can cause depressive and anxiety symptoms. (Eisenberger et al., 2010; Harrison et al., 2009) Interestingly, probiotics can reduce pro‐inflammatory cytokine levels (Ait‐Belgnaoui et al., 2012; Gareau, Silva, & Perdue, 2008; Luo et al., 2014) and oxidative stress (Liu & Zhu, 2018), increase anti‐inflammatory cytokine levels (Citar et al., 2015), and play an immune regulation role, silencing the inflammatory response. (Vitaliti, Pavone, Guglielmo, Spataro, & Falsaperla, 2014) Therefore, probiotic supplementations could help improve depressive and anxiety symptoms, leading to a general improvement of patients’ quality of life. (Peirce & Alviña, 2019).

Briefly, probiotics are living microorganisms whose intake in adequate quantities can prove beneficial for the host's health (Food & Agriculture Organization, 2001), producing neuroactive and neuroendocrine molecules, which also act on the central nervous system et al., 2009), and acting as immunomodulators by influencing cytokine secretion. (Thomas & Versalovic, 2010).

Animal and human studies have investigated the effects of probiotics, respectively, on anxiety‐like behavior and depressive‐like behavior in rats, (Arseneault‐Breard et al., 2012) and psychological dimensions in humans, with encouraging results. (Tillisch et al., 2013) Probiotic supplementations could be an optimal adjunct to conventional antidepressants in the treatment of depressive and anxiety symptoms. The mechanism by which probiotics achieve these effects is not completely elucidated, even though several hypotheses have been formulated. (Collins et al., 2009) Interestingly, an antimicrobial effect has been shown by antidepressants, which are widely acknowledged to act on serum cytokine levels as well. (Brunoni et al., 2014; Hannestad, DellaGioia, & Bloch, 2011; Macedo et al., 2017).

To consider probiotics as a viable option in the treatment of the major depressive disorder or other neuropsychiatric disorders, evidence from well‐defined clinical trials is needed; however, only a few clinical trials investigating the influence of probiotic consumption on behavior, mood, and cognition in the general population are available. In a previous meta‐analysis of ten randomized controlled trials, Ng, Peters, Ho, Lim, and Yeo (2018), Ng, Soh, Loke, Lim, and Yeo (2018)**, **have reported that the probiotic supplementation had overall insignificant effects on mood, with only modest effects in individuals with pre‐existing mood symptoms and insignificant effects in healthy, community‐dwelling individuals. According to this meta‐analysis, the efficacy of probiotics consumption on the improvement of depression and anxiety symptoms, quality of life, and inflammatory biomarkers still needs to be demonstrated.

### 1.1 Aims of the study

The aim of this review was to identify published data from randomized controlled trials (RCTs), studying the efficacy of probiotics consumption on the improvement of depressive symptoms, anxiety symptoms, quality of life, and inflammatory biomarkers. Another aim was the identification of the population which can maximally benefit from the probiotic treatment.

## 2 MATERIALS AND METHODS

A scoping review was conducted following the PRISMA‐ScR (PRISMA extension for Scoping Reviews), (Tricco et al., 2018) as reported in Checklist 1. The PubMed and Scopus databases were searched on September 15th, 2019, using the following keywords:

PubMed: (("depression") AND "inflammation") AND "probiotics"; Scopus: "depression AND probiotics" OR "depression AND inflammation" AND NOT INDEX (medline) AND (LIMIT‐TO (DOCTYPE, "ar") OR LIMIT‐TO (DOCTYPE, "re") OR LIMIT‐TO (DOCTYPE, "ch") OR LIMIT‐TO (DOCTYPE, "ip") OR LIMIT‐TO (DOCTYPE, "sh")) AND (LIMIT‐TO (LANGUAGE, "English")).

Two independent reviewers (E.G. and C.G.) assessed the articles identified by the above keywords.

After removing duplicates, titles were screened first, and those not in line with the purpose of the review were excluded. Then, abstracts were assessed, and last full texts were read, eventually leading to the inclusion or exclusion of the papers. The possible disagreement between reviewers was resolved by joint discussion with a third review author (P.Z.).

**FIGURE 1:**
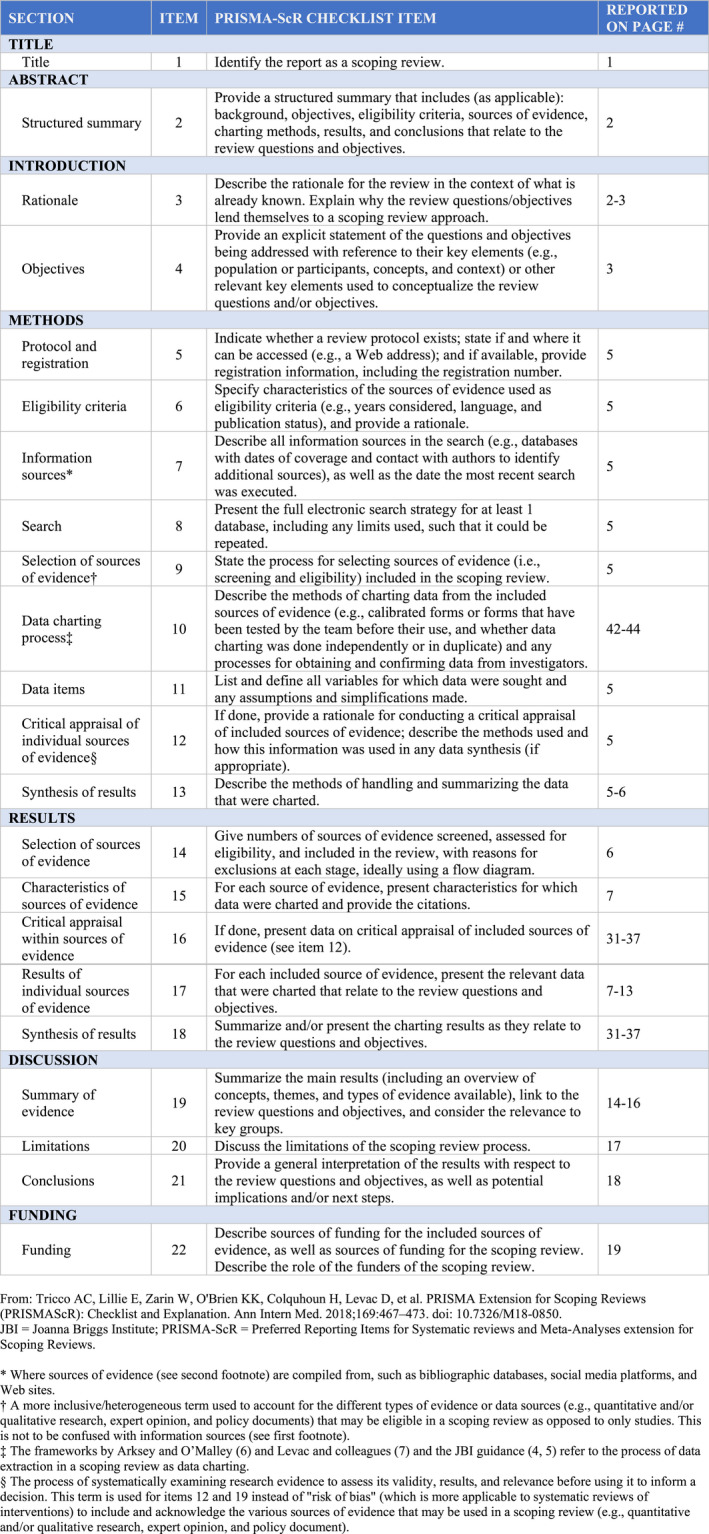
Preferred reporting items for systematic reviews and meta‐analyses extension for scoping reviews (PRISMA‐ScR) checklist

The consultation of an expert in this field of research allowed the inclusion of further 13 articles related to the topic and consistent with the search strings and the purpose of the study (as reported in Figure 1).

To be included in the review, studies had to: (a) deal with depression, inflammation, and probiotic supplementation; (b) be conducted on human beings (randomized controlled clinical trials, case–control studies, and prospective studies); (c) be written in English; (d) evaluate the effects of interventions on at least one of the following outcomes: anxiety, depressive symptoms, quality of life (QoL), global functioning, social adaptation, exogenous stressors, and biomarkers.

Animal and laboratory studies, those in a language different from English, gray literature and reviews of the literature were excluded.

Data extracted from the selected studies were recorded in a datasheet using a standardized coding form, including the following categorical and numerical variables: general information about the study (author/s, year of publication, duration of the study, title, journal title, country, study type, sample size, number in the experimental group, number in the control group, and lost at follow‐up), participants’ information (age and diagnosis), treatment (type of probiotic), intervention information (number of weeks of assumption), outcome assessment (questionnaire used and type of biomarker), and results.

Descriptive statistics used frequencies and percentages in the case of qualitative variables and means, standard deviations (SDs), and maxima and minima in the case of quantitative variables. Group differences in categorical variables were evaluated using the chi‐squared test, and group differences in continuous variables were assessed using a *t* test. A *p *< .05 was considered statistically significant. Analyses were performed using STATA 15. (StataCorp, 2017).

## 3 RESULTS

**FIGURE 2:**
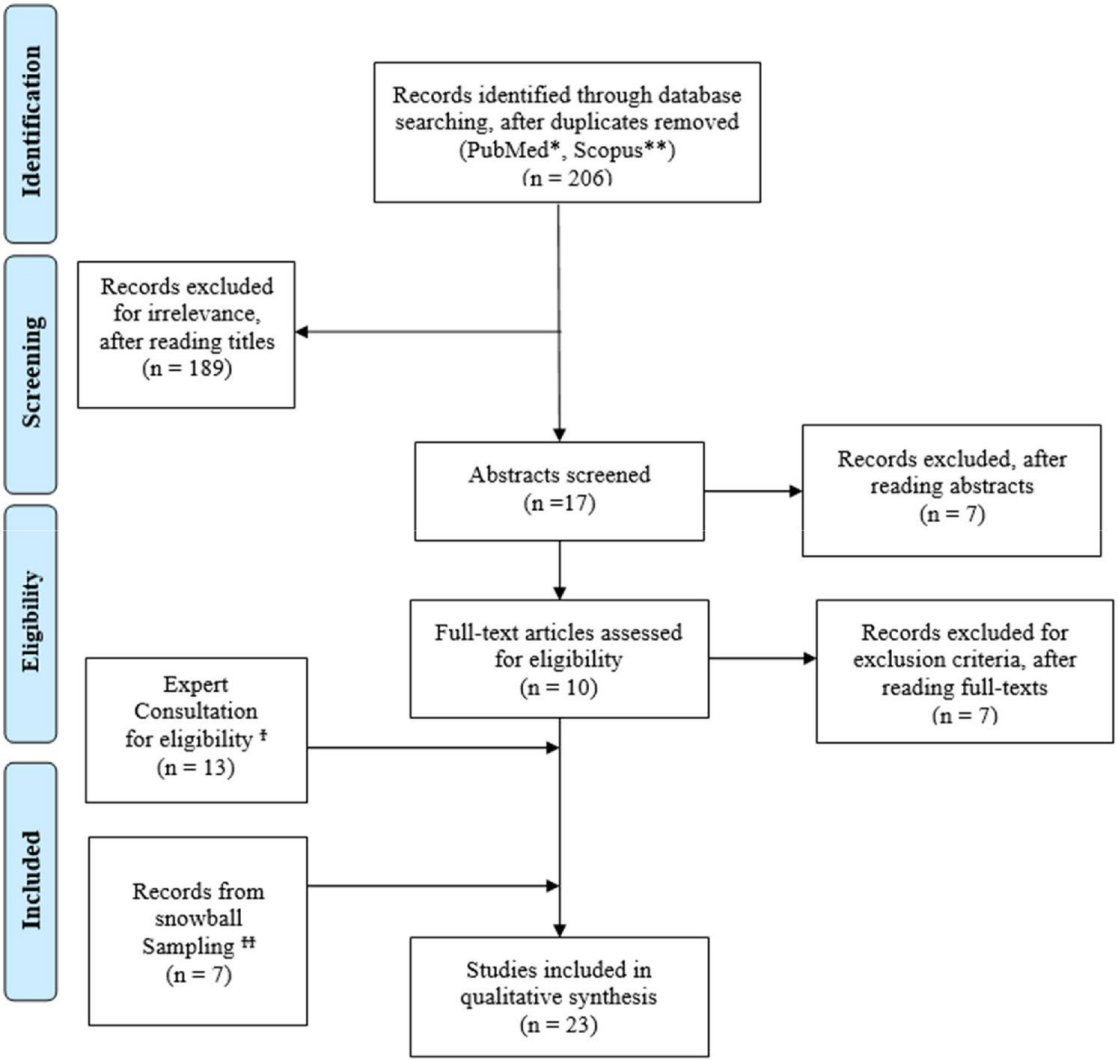
PRISMA flow chart

As described in the PRISMA flow diagram (Figure 2), the first search identified 206 titles; according to titles, 189 records were excluded; after reading the abstract, 7 further records were excluded: One study was excluded because it was an animal experimentation, and six studies because they were not clinical trials. Ten full texts were fully assessed for eligibility, and seven were excluded (5 were not clinical studies, and two studies did not include a probiotic supplementation). Furthermore, 13 records were included as suggested by expert consultation and 7 records were identified from two previous systematic reviews. The selection process eventually yielded 23 studies to be included in the review process. (Akkasheh et al., 2016; Begtrup, De Muckadell, Kjeldsen, Christensen, & Jarbol, 2013; Benton, Williams, & Brown, 2007; Chahwan et al., 2019; Feher et al., 2014; Guyonnet et al., 2007; Herranen et al., 2003; Hilimire, DeVylder, & Forestell, 2015; Kato‐Kataoka et al., 2016; Lorenzo‐Zúñiga et al., 2014; Lyra et al., 2016; Malaguarnera et al., 2012; Marcos et al., 2005; Messaoudi et al., 2011; Östlund‐Lagerström et al., 2016; Pinto‐Sanchez et al., 2017; Rao et al., 2009; Romijn, Rucklidge, Kuijer, & Frampton, 2017; Shinkai et al., 2013; Steenbergen, Sellaro, van Hemert, Bosch, & Colzato, 2015; Stevenson, Blaauw, Fredericks, Visser, & Roux, 2014; Tillisch et al., 2013; Vaghef‐Mehrabany et al., 2014).

The main features of the selected studies, including data on the first Author, country and year, patients’ features, probiotic treatment, outcomes and measures, and main findings, are shown in Table 1.

**TABLE 1:** Main features of studies included

Study		Country		Patients	Study type			Treatment		Intervention type		Intervention methodology		Outcomes And measures		Findings
(a) Main features of studies including patients with depression																
Akkasheh et al. (2016)		Iran		*n*: 40 *n* probiotic cases: 20 *n* placebo cases: 20 Mean age (probiotic group): 36.2 Mean age (placebo group): 38.3 Gender: mixed Diagnosis: MDD	Randomized, double‐blind, placebo‐controlled trial			NS		*L. acidophilus* (2 × 10^9^ CFU), *L. casei* (2 × 10^9^ CFU) and *B. bifidum* (2 × 10^9^ CFU).		Taking probiotic or placebo for 8 weeks		Depression: BDI; Biomarkers: blood sample		Reduction of depressive symptoms and insulin, HOMA‐IR, CRP and GSH serum levels
Chahwan et al. (2019)		Australia		*n*: 71 *n* probiotic cases: 34 *n* placebo cases: 37 Mean age (probiotic group): 36.65 Mean age (placebo group): 35.49 Gender: mixed Diagnosis: depression	Randomized, triple‐blind, placebo‐controlled trial			None		*B. bifidum* W23*, B.* *lactis* W51*, B. lactis* W52*, L. acidophilus* W37*, L. brevis* W63*, L. casei* W56*, L. salivarius* W24*, Lactococcus lactis* W19 and *Lactococcus lactis* W58 (1 x 10^10^ CFU/day)		Taking probiotic or placebo for 8 weeks		Anxiety: DASS−21, BAI Depression: MINI, DASS−21, BDI‐II, LEIDS‐R Biomarkers: fecal sample		No statistically significant effect of probiotic consumption on the outcomes assessed
Pinto‐Sanchez et al. (2017)		Canada		*n*: 44 *n* probiotic cases: 22 *n* placebo cases: 22 Mean age (probiotic group): 46.5 Mean age (placebo group): 40 Gender: mixed Diagnosis: IBS with HAD‐A or HAD‐D score between 8 and 14 (low–moderate depression)	Randomized, double‐blind, placebo‐controlled trial			None		*B. longum* NCC3001 (1.0E + 10 CFU)		Taking probiotic or placebo for 6 weeks		Anxiety: HADS‐A, STAI; Depression: HADS‐D; Quality of life: SF−36; Biomarkers: blood sample		Reduction of depression and quality of life improvement
Romijn et al. (2017)		New Zealand		*n*: 79 *n* probiotic cases: 40 *n* placebo cases: 39 Mean age (probiotic group): 35.8 Mean age (placebo group): 35.1 Gender: mixed Diagnosis: low–moderate depression	Randomized, double‐blind, placebo‐controlled trial			Psychotherapy		*L. helveticus* R0052 and *B. longum* R0175 (⩾3 × 10^9^ CFU/1.5 g sachet)		Taking probiotic or placebo for 8 weeks		Anxiety: DASS−42; Depression: MADRS, DASS−42, QIDS‐SR16, Global functioning: GAF; Biomarkers: blood sample		No statistically significant effect of probiotic consumption on the outcomes assessed
(b) Main features of studies involving patients with organic disease																
Begtrup et al. (2013)		Denmark		*n*: 132 *n* probiotic cases: 67 *n* placebo cases: 64 Mean age: 30.52 Gender: mixed Diagnosis: IBS	Randomized, double‐blind, placebo‐controlled trial			NS		*L. paracasei ssp paracasei* F19*, L. acidophilus* La5 and B. Bb12 (1.3 x 10^10^ CFU)		Taking probiotic or placebo for 6 months		Quality of life: HRQoL		No statistically significant effect of probiotic consumption on the outcomes assessed
Feher et al. (2014)		Hungary		*n*: 40 *n* probiotic cases: 20 *n* placebo cases: 20 Mean age (probiotic group): 45.5 Mean age (placebo group): 45.95 Gender: mixed Diagnosis: irritable eye syndrome	Prospective, open‐label Phase I/II controlled clinical trial			NS		*L. acidophilus* ATCC 4,356 (1.25 x 10^9^ CFU) and *B. longum* ATCC 15,707 (1.3 x 10^9^ CFU)		Taking probiotic or placebo for 8 weeks		Anxiety and depression: Irritable Eye Syndrome Testing Questionnaire for Diagnosis and Treatment Efficacy; Biomarkers: blood sample		Reduction of anxiety, depressive symptoms and inflammatory biomarkers
Guyonnet et al. (2007)		France		*n*: 276 *n* probiotic cases: 135 *n* placebo cases: 132 Mean age (probiotic group): 49.4 Mean age (placebo group): 49.2 Gender: mixed Diagnosis: IBS	Randomized, double‐blind, placebo‐controlled trial			NS		*B. animalis* DN−173 010 (1.25 x 10^10^ CFU), *S. thermophilus* and *L. bulgaricus* (1.2 x 10^9^ CFU)		Taking probiotic or placebo for 6 weeks		Anxiety, quality of life, global functioning: HRQoL		No statistically significant difference between the two groups. Reduction of anxiety and improvement of global functioning
Hatakka et al. (2003)		Finland		*n*: 26 *n* probiotic cases: 8 *n* placebo cases: 13 Mean age (probiotic group): 50 Mean age (placebo group): 53 Gender: mixed Diagnosis: rheumatoid arthritis	Randomized, double‐blind, placebo‐controlled trial			NS		*L. rhamnosus* (ATCC 53,103) GG (> 5 x 10^9^ CFU/capsule)		Taking probiotic or placebo, twice a day, for 12 months		Global functioning: HAQ; Biomarkers: blood sample		No statistically significant effect of probiotic consumption on the outcomes assessed
Lorenzo‐Zúñiga et al. (2014)		Spain		*n*: 84 *n* probiotic cases: 55 *n* placebo cases: 29 Mean age (high dose probiotic group): 47.5 Mean age (low‐dose probiotic group): 46.3 Mean age (placebo group): 46.5 Gender: mixed Diagnosis: IBS	Randomized, double‐blind, placebo‐controlled trial			None		*L. plantarum* (CECT7484 and CECT7485) and *P. acidilactici* (CECT7483) high dose (1–3 × 10^10^ CFU) and low dose (3–6 × 10^9^ CFU).		Taking probiotic or placebo for 6 weeks		Anxiety: VSI; Quality of life: HRQoL e IBS‐QoL		Statistically significant difference between the two groups
Lyra et al. (2016)		Finland		*n*: 391 *n* probiotic cases: 260 *n* placebo cases: 131 Mean age (high dose probiotic group): 47.2 Mean age (low‐dose probiotic group): 47.1 Mean age (placebo group): 49.9 Gender: mixed Diagnosis: IBS	Randomized, triple‐blind, placebo‐controlled trial			NS		*L. acidophilus* NCFM (ATCC 700,396) high dose (10^10^ CFU) and low dose (10^9^ CFU)		Taking probiotic or placebo for 12 weeks		Anxiety and depression: HADS; Quality of life: IBS‐QoL		Reduction of depression in the high dose group. No statistically significant difference between the two groups. Reduction of anxiety, no effects on quality of life.
Malaguarnera et al. (2012)		Italy		*n*: 66 *n* probiotic cases: 34 *n* placebo cases: 32 Mean age (probiotic group): 46.9 Mean age (placebo group): 46.7 Gender: mixed Diagnosis: NASH	Randomized, double‐blind, placebo‐controlled trial			NS		*B. longum* and FOS		Taking probiotic or placebo for 24 weeks		Biomarkers: blood sample		Reduction of CRP, HOMA‐IR, TNF‐α, Fasting Plasma Glucose
Rao et al. (2009)		USA		*n*: 39 *n* probiotic cases: 19 *n* placebo cases: 16 Mean age: NS Gender: mixed Diagnosis: chronic fatigue syndrome	Randomized, double‐blind, placebo‐controlled trial			NS		*L. casei strain Shirota* (8 x 10^9^ CFU/sachet)		Taking probiotic or placebo, three times a day, for 8 weeks		Anxiety: BAI; Depression: BDI		Reduction of anxiety
Stevenson et al. (2014)		South Africa		*n*: 81 *n* probiotic cases: 54 *n* placebo cases: 27 Mean age (probiotic group): 48.5 Mean age (placebo group): 47.27 Gender: mixed Diagnosis: IBS	Randomized, double‐blind, placebo‐controlled trial			NS		*L. plantarum* 299 v (5 x 10^9^ CFU)		Taking probiotic or placebo for 12 weeks		Quality of life: IBS‐QoL		No statistically significant effect of probiotic consumption on the outcomes assessed
Vaghef‐Mehrabany et al. (2014)		Iran		*n*: 46 *n* probiotic cases: 22 *n* placebo cases: 24 Mean age (probiotic group): 41.14 Mean age (placebo group): 44.29 Gender: female Diagnosis: rheumatoid arthritis	Randomized, double‐blind, placebo‐controlled trial			Metrotrexate, hydroxychloroquine, prednisolone.		*Lactobacillus casei* 01 (10^8^ CFU)		Taking probiotic or placebo for 8 weeks		Anxiety: STAI‐Y; Global functioning: Assess Global Health; Biomarkers: blood sample		Reduction of inflammatory biomarkers
(c) Main features of studies involving healthy subjects																
Benton et al. (2007)	England		*n*: 138 *n* probiotic cases: 66 *n* placebo cases: 66 Mean age: 61.8 Gender: mixed Diagnosis: none			Randomized, double‐blind, placebo‐controlled trial	None		*L. casei Shirota* (6.5 x 10^9^ CFU)		Taking probiotic or placebo for 3 weeks		Depression: POMS		Reduction of depressive symptoms	
Hilimire et al. (2015)	USA		*n*: 710 Mean age: 19.1 Gender: mixed Diagnosis: none			cross‐sectional approach	None		Probiotic foods		Taking probiotic		Anxiety: SPAI−23; Global functioning: Big Five Personality Inventory		Reduction of anxiety and improvement of global functioning	
Kato‐Kataoka et al. (2016)	Japan		*n*: 57 *n* probiotic cases: 24 *n* placebo cases: 23 Mean age (probiotic group): 23 Mean age (placebo group): 22.7 Gender: mixed Diagnosis: none			Randomized, double‐blind, placebo‐controlled trial	None		*L. casei strain Shirota* (1.0 × 10^9^ CFU/ml)		Taking probiotic or placebo for 8 weeks		Anxiety: STAI; Depression: HADS‐D, SDS. Biomarkers: blood, salivary and fecal sample		No statistically significant difference between the two groups. Reduction of fecal serotonin level	
Marcos et al. (2005)	Spain		*n*: 155 *n* probiotic cases: 73 *n* placebo cases: 63 Mean age: NS Gender: mixed Diagnosis: none			Prospective, Randomized, Controlled and parallel trial	None		*L. delbrueckii spp. Bulgaricus* (10^7^ CFU/mL) and *S. salivarius spp. thermophilus* (10^8^ CFU/mL) and *L. casei*		Taking probiotic or placebo for 6 weeks		Anxiety: STAI; Biomarkers: blood sample		Reduction of anxiety and white blood cells	
Messaoudi et al. (2011)	USA		*n*: 66 *n* probiotic cases: 28 *n* placebo cases: 28 Mean age (probiotic group): 42.4 Mean age (placebo group): 43.2 Gender: mixed Diagnosis: none			Randomized, double‐blind, placebo‐controlled trial	None		*L. helveticus* R0052 and *B. longum* R0175 (3 x 10^9^ CFU)		Taking probiotic or placebo for 4 weeks		Anxiety: HADS‐A, HSCL−90; Depression: HADS‐D, HSCL−90; Stressor: Perceived Stress Scale; Biomarkers: urine sample		Reduction of depression and free urinary cortisol	
Östlund‐Lagerström et al. (2016)	Sweden		*n*: 290 *n* probiotic cases: 143 *n* placebo cases: 147 Mean age: 73.1 Gender: mixed Diagnosis: none			Randomized, double‐blind, placebo‐controlled trial	NSAID, antihypertensives, PPI, opiates, OTC.		*L. reuteri* DSM 17,938 (1 × 10^8^ CFU)		Taking probiotic or placebo for 12 weeks		Anxiety and depression: HADS, Quality of life: EQ−5D−5L; Stressor: Perceived Stress Scale		No statistically significant effect of probiotic consumption on the outcomes assessed	
Shinkai et al. (2013)	Japan		*n*: 300 *n* probiotic cases: 200 *n* placebo cases: 100 Mean age (high dose probiotic group): 70.8 Mean age (low‐dose probiotic group):71 Mean age (placebo group): 70.9 Gender: mixed Diagnosis: none			Randomized, double‐blind, placebo‐controlled trial	None		*L. pentosus strain* b240 high dose (2 x 10^10^ CFU) and low dose (2 x 10^9^ CFU)		Taking probiotic or placebo for 20 weeks		Quality of lifeQualità di vita: SF−36		Quality of life improvement	
Steenbergen et al. (2015)	Netherlands		*n*: 40 *n* probiotic cases: 20 *n* placebo cases: 20 Mean age (probiotic group): 20.2 Mean age (placebo group): 19.7 Gender: mixed Diagnosis: none			Randomized, triple‐blind, placebo‐controlled trial	None		*B. bifidum* W23, *B. lactis* W52, *L. acidophilus* W37, *L. brevis* W63, *L. casei* W56, *L. salivarius* W24, and *Lactococcus lactis* (W19 e W58) (2.5 x 10^9^ CFU/g in 2 g sachet)		Taking probiotic or placebo for 4 weeks		Anxiety: BAI; Depression: BDI‐II e LEIDS‐R		Reduction of depression	
Tillisch et al. (2013)	USA		*n*: 36 *n* probiotic cases: 12 *n* placebo cases: 11 *n* control cases: 13 Mean age: 30 Gender: female Diagnosis: none			Randomized, double‐blind, placebo‐controlled trial	None		*B. animalis spp. lactis* (I−2494; 1.25 × 10^10^ CFU), *S. thermophilus* (CNCM I−1630; 1.2 × 10^9^ CFU) e *L. bulgaricus* (CNCM I−1632 e I−1519; 1.2 × 10^9^ CFU), and *Lactococcus lactis spp. lactis* (CNCM I−1631)		Taking probiotic or placebo, twice a day, for 4 weeks		Anxiety and depression: MINI Plus Biomarkers: blood sample		No statistically significant effect of probiotic consumption on the outcomes assessed	

Abbreviations: BAI, Beck Anxiety Inventory; BDI, Beck Depression Inventory; CFU, Colony‐forming Unit CRP, C‐reactive Protein;; DASS, Depression, Anxiety and Stress Scale; EQ‐5D‐5L, EuroQoL Dimensions and 5 Levels Measure of Health and Wellbeing; FOS, Fructooligosaccharides; GAF, Global Assessment of Functioning; GSH, Glutathione; HADS‐A, Hospital Anxiety and Depression Scale—Anxiety; HADS‐D, Hospital Anxiety and Depression Scale—Depression; HAQ, Health Assessment Questionnaire; HOMA‐IR, Homeostatis Model Assessment of Insulin Resistance; HRQoL, Health‐related Quality of Life; HSCL‐90, Hopkins Symptoms Checklist—90; IBS, Irritable Bowel Syndrome; IBS‐QoL, Irritable Bowel Syndrome—Quality of Life; LEIDS‐R, Leiden Index of Depression Sensitivity—R; MADRS, Montgomery–Asberg Depression Rating Scale; MDD, Major Depressive Disorder; MINI, Mini International Neuropsychiatric Interview; NASH, Nonalcoholic Steatosis Hepatitis; NS, Not Specified; NSAID, Nonsteroidal Anti‐inflammatory Drugs; OTC, Over The Counter; POMS, Profile of Mood States; PPI, Proton‐pump Inhibitors; QIDS‐SR16, Quick Inventory of Depressive Symptomatology, 16 Items, Self‐report; SDS, standard deviations; SF‐36, Short Form Health Survey—36; SPAI‐23, Social Phobia and Anxiety Inventory—23; STAI‐Y, State Trait Anxiety Inventory—Y; TNF, Tumor Necrosis Factor; VSI, Visceral Sensitivity Index.

### 3.1 General information

Most of the selected studies (*N* = 17) (73.9%) were randomized, double‐blind, placebo‐controlled trials. (Akkasheh et al., 2016; Begtrup et al., 2013; Benton et al., 2007; Guyonnet et al., 2007; Herranen et al., 2003; Kato‐Kataoka et al., 2016; Lorenzo‐Zúñiga et al., 2014; Malaguarnera et al., 2012; Messaoudi et al., 2011; Östlund‐Lagerström et al., 2016; Pinto‐Sanchez et. al, 2017; Rao et al., 2009; Romijn et al., 2017; Shinkai et al., 2013; Stevenson et al., 2014; Tillisch et al., 2013; Vaghef‐Mehrabany et al., 2014) We included also 3 (13%) randomized, triple‐blind, placebo‐controlled trials (Chahwan et al., 2019; Lyra et al., 2016; Steenbergen et al., 2015), one (4.3%) prospective, randomized, controlled, and parallel trial, (Marcos et al., 2005) one (4.3%) prospective, open‐label phase I/II controlled clinical trial (Feher et al., 2014), and one (4.3%) cross‐sectional approach. (Hilimire et al., 2015) Only one study (4.3%) did not use randomization. (Hilimire et al., 2015).

Most studies lasted a few weeks: 12 weeks in 3 studies (13%), (Lyra et al., 2016; Östlund‐Lagerström et al., 2016; Stevenson et al., 2014) 8 weeks in 7 studies (30.1%), (Akkasheh et al., 2016; Chahwan et al., 2019; Feher et al., 2014; Kato‐Kataoka et al., 2016; Rao et al., 2009; Romijn et al., 2017; Vaghef‐Mehrabany et al., 2014) 6 weeks in 4 studies (17.4%), (Guyonnet et al., 2007; Lorenzo‐Zúñiga et al., 2014; Marcos et al., 2005; Pinto‐Sanchezet al., 2017) 4 weeks in 3 studies (13%), and (Messaoudi et al., 2011; Steenbergen et al., 2015; Tillisch et al., 2013) 3 weeks in only one study (4.3%). (Benton et al., 2007) 2 studies (8.7%) lasted 24 (Malaguarnera et al., 2012) and 20 (Shinkai et al., 2013) weeks, respectively. Nonetheless, there were also 2 studies (8.7%) with a much longer duration (52 weeks). (Begtrup et al., 2013; Herranen et al., 2003) The information about the duration of the trial was not specified in one study only (4.3%). (Hilimire et al., 2015).

In all studies, a follow‐up was performed. One study (4.3%) set a single follow‐up visit, (Hilimire et al., 2015) while 8 studies (34.8%) performed two visits, (Akkasheh et al., 2016; Chahwan et al., 2019; Feher et al., 2014; Marcos et al., 2005; Rao et al., 2009; Romijn et al., 2017; Steenbergen et al., 2015; Vaghef‐Mehrabany et al., 2014) and 6 studies (26%) three visits. (Benton et al., 2007; Guyonnet et al., 2007; Lorenzo‐Zúñiga et al., 2014; Messaoudi et al., 2011; Östlund‐Lagerström et al., 2016; Tillisch et al., 2013) Three studies (13%) proposed four visits (Begtrup et al., 2013; Lyra et al., 2016; Pinto‐Sanchezet al., 2017), 2 (8.7%) five visits (Herranen et al., 2003; Kato‐Kataoka et al., 2016), 2 six visits (8,16), and one (4.3%) seven visits (Shinkai et al., 2013) after the beginning of the intervention.

The studies were published in several countries all over the world; however, they were mostly from the United States (*N* = 4) (17.4%). (Hilimire et al., 2015; Messaoudi et al., 2011; Rao et al., 2009; Tillisch et al., 2013;3) Concerning the period of publication, 11 studies (47.8%) were published between 2011 and 2015 (Akkasheh et al., 2016; Begtrup et al., 2013; Feher et al., 2014; Hilimire et al., 2015; Lorenzo‐Zúñiga et al., 2014; Malaguarnera et al., 2012; Shinkai et al., 2013; Steenbergen et al., 2015; Stevenson et al., 2014; Tillisch et al., 2013; Vaghef‐Mehrabany et al., 2014), 6 in the 2016–2019 period (Chahwan et al., 2019; Kato‐Kataoka et al., 2016; Lyra et al., 2016; Östlund‐Lagerström et al., 2016; Pinto‐Sanchez et al., 2017; Romijn et al., 2017), 4 between 2006 and 2010 (Benton et al., 2007; Guyonnet et al., 2007; Messaoudi et al., 2011; Rao et al., 2009), and 2 before 2005. (Herranen et al., 2003; Marcos et al., 2005).

Only one of the studies did not specify the number of centers involved. (Lorenzo‐Zúñiga et al., 2014) Nineteen (82.6%) were monocentric (Akkasheh et al., 2016; Begtrup et al., 2013; Benton et al., 2007; Chahwan et al., 2019; Feher et al., 2014; Hilimire et al., 2015; Kato‐Kataoka et al., 2016; Malaguarnera et al., 2012; Marcos et al., 2005; Messaoudi et al., 2011; Pinto‐Sanchez et al., 2017; Östlund‐Lagerström et al., 2016; Rao et al., 2009; Romijn et al., 2017; Shinkai et al., 2013; Steenbergen et al., 2015; Stevenson et al., 2014; Tillisch et al., 2013; Vaghef‐Mehrabany et al., 2014), 2 (8.7%) involved two centers (Herranen et al., 2003; Lyra et al., 2016), and one study (4.3%) 35 centers. (Guyonnet et al., 2007) All studies except one used a placebo, (4.3%). (Hilimire et al., 2015).

Seven studies (30.4%) were performed in a university setting (Chahwan et al., 2019; Kato‐Kataoka et al., 2016; Lorenzo‐Zúñiga et al., 2014; Östlund‐Lagerström et al., 2016; Rao et al., 2009; Romijn et al., 2017; Shinkai et al., 2013), 5 (21.7%) in a hospital setting (Akkasheh et al., 2016; Herranen et al., 2003; Pinto‐Sanchez et al., 2017; Tillisch et al., 2013; Vaghef‐Mehrabany et al., 2014), and 4 (17.4%) in a medical office. (Begtrup et al., 2013; Chahwan et al., 2019; Feher et al., 2014; Marcos et al., 2005).

### 3.2 Participants’ features

The selected studies involved different populations: 9 (39.1%) were performed on a sample from the general population, (Benton et al., 2007; Hilimire et al., 2015; Kato‐Kataoka et al., 2016; Marcos et al., 2005; Messaoudi et al., 2011; Östlund‐Lagerström et al., 2016; Shinkai et al., 2013; Steenbergen et al., 2015; Tillisch et al., 2013;) 5 (21.7%) on inflammatory bowel syndrome (IBS)‐affected population (Begtrup et al., 2013; Guyonnet et al., 2007; Lorenzo‐Zúñiga et al., 2014; Lyra et al., 2016; Stevenson et al., 2014), and 2 studies (8.7%) considered patients with the comorbidity IBS‐Depression. (Pinto‐Sanchez et al., 2017; Romijn et al., 2017) Overall, the population involved suffered from a IBS syndrome in 30.4% of cases (*N* = 7). (Begtrup et al., 2013; Guyonnet et al., 2007; Lorenzo‐Zúñiga et al., 2014; Lyra et al., 2016; Pinto‐Sanchez et al., 2017; Romijn et al., 2017; Stevenson et al., 2014).

Gender was mixed in all studies except 2 (8.7%) that considered only a female population. (Tillisch et al., 2013; Vaghef‐Mehrabany et al., 2014) The ethnicity of participants was not specified in most cases (*N* = 17) (73.9%), (Akkasheh et al., 2016; Benton et al., 2007; Feher et al., 2014; Guyonnet et al., 2007; Herranen et al., 2003; Kato‐Kataoka et al., 2016; Lorenzo‐Zúñiga et al., 2014; Lyra et al., 2016; Malaguarnera et al., 2012; Marcos et al., 2005; Östlund‐Lagerström et al., 2016; Shinkai et al., 2013; Steenbergen et al., 2015; Stevenson et al., 2014; Tillisch et al., 2013; Vaghef‐Mehrabany et al., 2014) while in 4 (17.4%) (Chahwan et al., 2019; Hilimire et al., 2015; Pinto‐Sanchez et al., 2017; Romijn et al., 2017) and 2 (8.7%) studies it was mixed and Caucasian, respectively. (Begtrup et al., 2013; Messaoudi et al., 2011).

Twelve studies (52.1%) did not specify details about the possible psychiatric diagnosis of the population assessed; (Begtrup et al., 2013; Guyonnet et al., 2007; Herranen et al., 2003; Hilimire et al., 2015; Lyra et al., 2016; Malaguarnera et al., 2012; Marcos et al., 2005; Östlund‐Lagerström et al., 2016; Rao et al., 2009; Shinkai et al., 2013; Stevenson et al., 2014; Vaghef‐Mehrabany et al., 2014) however, 3 studies (13%) indicated the presence of depression or anxiety in the sample, (Chahwan et al., 2019; Pinto‐Sanchez et al., 2017; Romijn et al., 2017) and one (4.3%) of major depressive disorder (MDD). (Akkasheh et al., 2016) The severity of depression was evaluated only in 3 works (13%) (Akkasheh et al., 2016; Pinto‐Sanchez et al., 2017): 2 (8.7%) identified low‐moderate depression (Pinto‐Sanchez et al., 2017; Romijn et al., 2017) and one severe depression in the population analyzed. (Akkasheh et al., 2016) In the studies where a psychiatric diagnosis was reported, patients were not under any pharmacological treatment; (Pinto‐Sanchez et al., 2017; Romijn et al., 2017) only psychotherapy was mentioned by one study (4.3%), (Romijn et al., 2017) and in the case of severe depression (Akkasheh et al., 2016) no information about treatment was provided.

The presence of treatment‐related adverse events was not specified by most studies (*N* = 11) (47.8%), (Akkasheh et al., 2016; Benton et al., 2007; Guyonnet et al., 2007; Herranen et al., 2003; Hilimire et al., 2015; Kato‐Kataoka et al., 2016; Marcos et al., 2005; Messaoudi et al., 2011; Shinkai et al., 2013; Steenbergen et al., 2015; Tillisch et al., 2013); among those that specified this data, no adverse event was reported by 7 studies (*N* = 7) (30.4%). (Begtrup et al., 2013; Feher et al., 2014; Lorenzo‐Zúñiga et al., 2014; Östlund‐Lagerström et al., 2016; Pinto‐Sanchez et al., 2017; Rao et al., 2009; Vaghef‐Mehrabany et al., 2014), while 5 specified the presence of adverse events. (Lyra et al., 2016; Malaguarnera et al., 2012; Östlund‐Lagerström et al., 2016; Romijn et al., 2017; Stevenson et al., 2014).

### 3.3 Outcomes

Studies included in the analysis used different questionnaires, either self‐reported or clinician‐rated, to evaluate different outcomes (Table 2). Several studies did not specify details about this information.

**TABLE 2:** Frequency distribution of outcome‐related qualitative variables

	*N*	%
DEPRESSION		
Evaluation of the effects of probiotics on depression (23/23)		
Yes	13	56.55
No	10	43.5
TOT	23	100
Measure (13/23)		
BDI	2	15.38
BDI‐II e LEIDS‐R	1	7.69
HADS‐D	1	7.69
HADS‐D e HSCL−90	1	7.69
HADS	4	30.76
MADRS, DASS−42 e QIDS‐SR16	1	7.69
Irritable eye syndrome testing questionnaire for diagnosis and treatment efficacy	1	7.69
MINI plus	1	7.69
POMS	1	7.69
TOT	13	100
Statistically significant reduction of depression levels (13/23)		
Yes	7	53.83
No	6	46.14
TOT	13	100
ANXIETY		
Evaluation of the effects of probiotics on anxiety (23/23)		
Yes	16	69.9
No	7	30.45
TOT	23	100
Measure (16/23)		
STAI	2	12.5
STAI‐Y	1	6.25
HADS‐A, HSCL−90	1	6.25
HADS‐A, STAI	1	6.25
HADS	2	12.5
HRQoL	1	6.25
BAI	2	12.5
DASS−42	1	6.25
DASS−42, BAI	1	6.25
Irritable eye syndrome testing questionnaire for diagnosis and treatment efficacy	1	6.25
MINI Plus	1	6.25
SPAI−23	1	6.25
VSI	1	6.25
TOT	16	100
Statistically significant reduction of anxiety levels (16/23)		
Yes	7	43.75
No	9	56.25
TOT	16	100
QUALITY OF LIFE		
Evaluation of the effects of probiotics on QoL (23/23)		
Yes	8	34.8
No	15	65.25
TOT	23	100
Measurement method (8/23)		
HRQoL	2	25
IBS‐QoL	2	25
HRQoL e IBS‐QoL	1	12.5
SF−36	2	25
EQ−5D−5L	1	12.5
TOT	8	100
Statistically significant improvement of QoL (8/23)		
Yes	3	37.5
No	5	62.5
TOT	8	100
GLOBAL FUNCTIONING		
Evaluation of the effects on the global functioning (23/23)		
Yes	5	21.75
No	18	78.3
TOT	23	100
Measurement method (5/23)		
HRQoL	1	20
GAF	1	20
HAQ	1	20
Big five personality inventory	1	20
Assess global health	1	20
TOT	5	100
Statistically significant improvement of the global functioning (5/23)		
Yes	2	40
No	3	60
TOT	5	100
BIOMARKERS		
Evaluation of the effects of probiotics on Biomarkers (23/23)		
Yes	12	52.2
No	11	47.85
TOT	23	100
Measures (12/23)		
Blood sample	9	74.97
Blood, salivary and fecal sample	1	8.33
Fecal sample	1	8.33
Urine sample	1	8.33
TOT	12	100
Statistically significant effects on Biomarkers (12/23)		
Yes	7	58.31
No	5	41.65
TOT	12	100

### 3.4 Depression

Thirteen (Kato‐Kataoka et al., 2016; Messaoudi et al., 2011; Östlund‐Lagerström et al., 2016; Pinto‐Sanchez et al., 2017; Rao et al., 2009; Romijn et al., 2017; Steenbergen et al., 2015; Tillisch et al., 2013) of the 23 studies included in our analysis considered the effect of probiotic consumption on the improvement of the depressive symptoms. Seven (Akkasheh et al., 2016; Benton et al., 2007; Feher et al., 2014; Lyra et al., 2016; Messaoudi et al., 2011; Pinto‐Sanchez et al., 2017; Steenbergen et al., 2015) out of these 13 studies reported a significant improvement of depressive symptoms after probiotic consumption, as measured by self‐rated and clinician‐rated questionnaires. Depression was measured as follows: with the Hamilton Anxiety Depression Scale (HADS) questionnaire by 4 studies, with the Beck Depression Inventory (BDI) by 2, and with different tools by the remaining.

Four (Akkasheh et al., 2016; Chahwan et al., 2019; Pinto‐Sanchez et al., 2017; Romijn et al., 2017) of these 13 studies included a population of depressed patients. One (Akkasheh et al., 2016) recruited a sample of MDD patients, while the others (Chahwan et al., 2019; Pinto‐Sanchez et al., 2017; Romijn et al., 2017) recruited patients with low–moderate depression. Only 2 studies (Akkasheh et al., 2016; Pinto‐Sanchez et al., 2017) supported a significant reduction of depressive symptoms.

Correlation analysis is described in Table 3. An association between probiotics efficacy in terms of reduction of depression was found only in studies where the sample did not include patients with psychiatric disorders (*p* = .03). No association was found among depression severity, the population involved, or type of probiotic.

**TABLE 3:** Correlation between independent variables and intervention efficacy in the improvement of the outcomes

Variables		DEPRESSION REDUCTION				ANXIETY REDUCTION				QoL IMPROVEMENT				BIOMARKERS REDUCTION		
		SE	NSE	NC	p	SE	NSE	NC	p	SE	NSE	NC	p	SE	NSE	p
Psychiatric diagnosis	Anxiety and/or depression	2	2	0	0.03*	0	3	1	0.1	1	0	3	0.33	2	2	0.46
	Nothing	4	2	1		2	4	1		1	0	6		3	4	
	NS	1	2	9		5	2	5		1	5	6		3	9	
	Total	7	6	10		7	9	7		3	5	15		8	15	
Depression severity	MDD	1	0	0	0.37	0	0	1	0.23	0	0	1	0.5	1	0	0.32
	Low–Moderate	1	1	0		0	2	0		1	0	1		1	1	
	NC	5	5	10		7	7	6		2	5	13		6	14	
	Total	7	6	10		7	9	7		3	5	15		8	15	
Probiotic species	Bifidobacteria	1	1	0	0.69	0	2	0	0.43	1	0	1	0.72	1	1	0.81
	Lactobacilla	2	3	5		3	3	4		1	3	6		3	7	
	Mixed	4	2	4		3	4	3		1	2	7		4	6	
	NS	0	0	1		1	0	0		0	0	1		0	1	
	Total	7	6	10		7	9	7		3	5	15		8	15	
Population involved	Depression	1	1	0	0.34	0	1	1	0.32	0	0	2	0.09	1	1	0.53
	General	3	3	3		2	5	2		1	1	7		3	6	
	IBS	0	0	4		2	0	2		1	3	0		0	4	
	IBS with Depression	1	1	0		0	2	0		1	0	1		1	1	
	Other	2	1	3		3	1	2		0	1	5		3	3	
	Total	7	6	10		7	9	7		3	5	15		8	15	

Abbreviations: NC, not considered; NS, not specified; NSE, nonsignificant efficacy; SE, significant efficacy.

### 3.5 Anxiety

Sixteen studies (Chahwan et al., 2019; Feher et al., 2014; Guyonnet et al., 2007; Kato‐Kataoka et al., 2016; Hilimire et al., 2015; Lorenzo‐Zúñiga et al., 2014; Lyra et al., 2016; Marcos et al., 2005; Pinto‐Sanchez et al., 2017; Messaoudi et al., 2011; Östlund‐Lagerström et al., 2016; Rao et al., 2009; Romijn et al., 2017; Steenbergen et al., 2015; Tillisch et al., 2013; Vaghef‐Mehrabany et al., 2014) evaluated the effects of probiotics on anxiety. The questionnaires used for anxiety assessment were not homogenous across studies. Seven out of these 16 studies (Feher et al., 2014; Guyonnet et al., 2007; Hilimire et al., 2015; Lorenzo‐Zúñiga et al., 2014; Lyra et al., 2016; Marcos et al., 2005; Rao et al., 2009) demonstrated an improvement of symptomatology.

No improvement of anxiety symptoms was reported by those 3 studies (Chahwan et al., 2019; Pinto‐Sanchez et al., 2017; Romijn et al., 2017) which recruited a population with low–moderate depression.

No significant result emerged from the correlation analysis between the reduction of anxiety symptoms and other variables.

### 3.6 Quality of life

Eight studies (Begtrup et al., 2013; Guyonnet et al., 2007; Lorenzo‐Zúñiga et al., 2014; Lyra et al., 2016; Pinto‐Sanchez et al., 201; Östlund‐Lagerström et al., 2016; Shinkai et al., 2013; Stevenson et al., 2014) analyzed QoL improvement after probiotic consumption, but only 3 of them (Lorenzo‐Zúñiga et al., 2014; Pinto‐Sanchez et al., 2017; Shinkai et al., 2013) demonstrated a significant effect after the intervention period. One of these studies (Pinto‐Sanchez et al., 2017) included a population with a diagnosis of low–moderate depression which showed a QoL improvement.

No significant result emerged from the correlation analysis between QoL improvement and other variables.

### 3.7 Global functioning

Five (Guyonnet et al., 2007; Herranen et al., 2003; Hilimire et al., 2015; Romijn et al., 2017; Vaghef‐Mehrabany et al., 2014) of the studies included in this review analyzed the improvement of global functioning in the population; 2 studies (Guyonnet et al., 2007; Hilimire et al., 2015) demonstrated a significant effect, associated with a reduction of anxiety symptoms, but none of them included a depressed population.

### 3.8 Biomarkers

More than half of the studies (*N* = 12) (Akkasheh et al., 2016; Chahwan et al., 2019; Feher et al., 2014; Herranen et al., 2003; Kato‐Kataoka et al., 2016; Malaguarnera et al., 2012; Marcos et al., 2005; Messaoudi et al., 2011; Pinto‐Sanchez et al., 2017; Romijn et al., 2017; Tillisch et al., 2013; Vaghef‐Mehrabany et al., 2014) evaluated the effects of probiotic intake on the reduction of inflammatory biomarkers. Ten out of these 12 studies analyzed blood samples, in one case (Kato‐Kataoka et al., 2016) in association with fecal and salivary samples, while one study (Messaoudi et al., 2011) assessed isolated urine sample, and another one (Chahwan et al., 2019) isolated fecal sample. Seven out of these 10 studies (Akkasheh et al., 2016; Feher et al., 2014; Kato‐Kataoka et al., 2016; Malaguarnera et al., 2012; Marcos et al., 2005; Messaoudi et al., 2011; Vaghef‐Mehrabany et al., 2014) demonstrated a significant effect of probiotics on biomarkers.

All of the studies recruiting patients with a diagnosis of depression (Akkasheh et al., 2016; Chahwan et al., 2019; Pinto‐Sanchez et al., 2017) analyzed inflammatory biomarkers after the probiotic treatment, but only one of them, (Akkasheh et al., 2016) which included a population with a diagnosis of MDD, demonstrated an improvement of some inflammation‐related parameters and insulin metabolism.

No significant result emerged from the correlation analysis between the improvement of inflammatory biomarkers and other variables.

## 4 DISCUSSION

In the current literature, the number of clinical studies evaluating the impact of probiotic supplementation on anxiety and depressive symptoms, QoL, and inflammatory biomarkers remain limited. Furthermore, these studies do not follow a standardized methodology.

Only in the last years probiotic integration caught the attention of the scientific community; hence, the effects of the alteration of the intestinal microbiota and the mechanisms underlying its role in various medical disorders still need to be clarified.

### 4.1 General features of the studies

In many studies in this research field, an important source of variability is the choice of the target population. Some studies focus on patients with chronic conditions, such as IBS, (Begtrup et al., 2013; Guyonnet et al., 2007; Lorenzo‐Zúñiga et al., 2014; Lyra et al., 2016; Pinto‐Sanchez et al., 2017; Stevenson et al., 2014) which can lead to mood changes, while others involve a healthy population, without clinical symptoms. (Benton et al., 2007; Hilimire et al., 2015; Kato‐Kataoka et al., 2016; Marcos et al., 2005; Messaoudi et al., 2011; Östlund‐Lagerström et al., 2016; Shinkai et al., 2013; Steenbergen et al., 2015; Tillisch et al., 2013).

Previous studies have highlighted that in IBS are present subclinical inflammation at the gut mucosa level as well as the involvement of psychosocial factors. (Ng, Soh, et al., 2018) Probiotics could be potentially useful in this setting as it has alleged anti‐inflammatory and immunomodulatory effects.

Various questionnaires, both self‐administered and clinician‐rated, were used for the assessment of outcomes and clinical variables: They considered different items (Julian, 2011) and had different psychometric properties. The present review did not apply restrictions on the questionnaires in order not to excessively limit the number of the studies included.

Regarding inflammatory biomarkers, the studies selected for this review showed variability in those assessed and also in the biological samples collected. Moreover, the difference in sample size across studies could influence the possibility to compare their results.

The probiotic supplementation in the various studies presented two further elements of variability: the duration of administration (from several weeks to several months) and the composition. This could be relevant in the comparison of the results since it is acknowledged the species specificity of the effects of probiotics in the treatment of different medical conditions. ( Bercik et al., 2010) The current literature is not well equipped to answer questions on the safety of probiotic interventions with confidence as there appears to be a lack of systematic reporting of adverse events. (Gwee et al., 2018). The available evidence does not indicate an increased risk, but there are anecdotal reports that probiotics may worsen outcomes, for example, in patients receiving radiotherapy (Hempel et al., 2011).

In the current scoping review, all the studies reported that probiotic treatment was well tolerated, with no relevant side effects.

It is important to underline that not all probiotics are equal. The Human Microbiome project revealed the microbial taxa complexity in the human gut, and also highlighted the highly individualized microbiome composition due to inheritance, diet, and environmental factors. Every effort should be made to report specific probiotic strains or mixture of strains when analyzing the efficacy and safety of probiotics (McFarland, Evans, & Goldstein, 2018).

It is also important to highlight that there are still existing gaps in knowledge regarding the interaction between the microbiome and the host in vivo—and the pathway of its metabolites—and how their metabolites influence the microenvironment. Further mechanistic studies involving "omics" technologies, as adapted from previous studies (Wang et al., 2018), might help shed light on these questions.

### 4.2 Outcomes

#### 4.2.1 Depression and anxiety

The impact of probiotic supplementation was described as effective in reducing depressive symptoms and anxiety by 53.83% and 43.75% of the studies, and in improving QoL and global functioning by 37.5% and 40% of the studies, respectively. Currently, only a few studies are available that focus on patients with depression, without any further comorbidity, and only one study (Akkasheh et al., 2016) has involved patients with a MDD diagnosis. Even in this case, anyway, no comparison with populations affected by subthreshold depression or Healthy Controls (HCs) was made; however, in all the populations examined, data concerning the improvement of QoL and depressive and anxious symptoms were analyzed.

These results seem to be in accordance with those from a previous review conducted by Ng, Soh, et al. (2018), who described no significant difference in mood between the treatment and placebo group postintervention, even if significant improvements were observed in the mood of individuals with mild to moderate depressive symptoms, and nonsignificant effects in healthy individuals.

The use of probiotics was effective in reducing depressive symptoms in 50% of the studies conducted on patients with depression in comorbidity with IBS. (Pinto‐Sanchez et al., 2017; Rao et al., 2009).

In patients affected by IBD, changes in the inflammatory biomarkers after probiotic supplementation were not statistically significant: further studies in this population would be necessary because of the strong impact on quality of life and on the onset of depressive symptoms. (Chey, Kurlander, & Eswaran, 2015; Dinan et al., 2006; Liebregts et al., 2007; Longstreth et al., 2006; Whorwell, McCallum, Creed, & Roberts, 1986).

#### 4.2.2 Biomarkers

Significant results have been reported by 58.31% of studies evaluating changes in inflammatory biomarkers, which is encouraging.

Considering the few studies that included a population with a diagnosis of depression, (Akkasheh et al., 2016; Chahwan et al., 2019; Pinto‐Sanchez et al., 2017; Romijn et al., 2017) inflammatory biomarkers were significantly reduced only in the study that considered a population with MDD: (Akkasheh et al., 2016) This result is consistent with the inflammatory hypothesis of depression.

#### 4.2.3 Quality of life and global functioning

In the literature, it has been shown that the microbiota can influence the CNS functions, (Martin‐Subero, Anderson, Kanchanatawan, Berk, & Maes, 2016) including mood regulation; hence, the possibility of acting directly on the microbiota using probiotic formulations with species‐specific effects (Liu & Zhu, 2018; Mangiola et al., 2016) to achieve mood changes and, consequently, an improvement in the quality of life and global functioning.

Only three (Lorenzo‐Zúñiga et al., 2014; Pinto‐Sanchez et al., 2017; Shinkai et al., 2013) of the eight studies considering the impact of probiotic integration in quality of life showed a significant improvement of this variable.

### 4.3 Strengths and limitations

The current review could add to the existing literature on the use of probiotic supplementation in the treatment of mood and anxiety disorders or symptoms, which is still lacking methodologically sound clinical studies and systematic reviews. The use of a standardized methodological protocol, the PRISMA statement, (Moher, Liberati, Tetzlaff, Altman, & PRISMA Group, 2009) is a strength of the current review.

Some limitations should be underscored. First, we have included only 23 studies, identified through two databases only: PubMed and Scopus. Second, the literature is still lacking clinical studies about the topic of probiotic integration and its impact on depression, anxiety, and QoL. Another limitation of this scoping review is that we did not contact the study authors to provide additional data, but other articles were read in which the methodology of the included studies was explained; furthermore, we did not search the gray literature.

Moreover, possible psychotherapeutic support was not considered in studies examined, which could be fundamental in reducing depressive (even subthreshold) and anxious symptomatology. (Cuijpers, Huibers, Ebert, Koole, & Andersson, 2013; Driessen, Cuijpers, Hollon, & Dekker, 2010; Williams et al., 1999) Finally, the available studies are poorly consistent in approach and methodology, making it difficult to generalize their results.

## 5 CONCLUSION

Our review found that available literature on this topic is very heterogeneous regarding type of probiotic used and duration of treatment, type of sample, methodology, assessment tools, and outcomes. Therefore, it is still difficult to draw clear conclusions about the effectiveness of probiotic supplementation in patients with depression and anxiety symptoms.

The number of clinical studies that examine probiotic supplementation in patients with depression is still limited, (Akkasheh et al., 2016; Chahwan et al., 2019; Pinto‐Sanchez et al., 2017) but they have shown promising, even though preliminary, results. Further studies with a sound and consistent methodological approach and more extensive meta‐analyses are warranted to support the results available in the existing literature about the potential benefit of probiotic supplementation in patients with major and subthreshold depression.

### Summations

(a). The concept of “gut–brain axis” is of great interest for the current research, and it has been suggested the hypothesis that probiotic treatment could improve depressed patients’ symptoms and inflammatory status. (b). Many trials have been performed about the effects of probiotic intake on depressive symptoms and inflammatory biomarkers with promising results, even though only few of them have actually included a sample of patients diagnosed with depression. (c). For these reasons, other trials and reviews are needed to increase knowledge in this field of research.

### Limitations

(a). One of the main limitations of this review is the lack of studies including a population affected by depression. (b). For this reason, we could include only 23 studies in our review, identified by two databases only. (c). Furthermore, the studies included are heterogeneous regarding the type of probiotic, the methods used to test symptoms and inflammatory status, and study outcomes; for these reasons, the possibility to analyze and generalize the emerging results is limited.

## CONFLICT OF INTERESTS

The authors declare that they have no competing interests.

## AUTHORS’ CONTRIBUTIONS

Eleonora Gambaro, Carla Gramaglia, and Patrizia Zeppegno contributed to the conception and design of the work; Carla Gramaglia and Patrizia Zeppegno developed and implemented the methods of this manuscript; Giulia Baldon, Emilio Chirico, Maria Martelli, and Alessia Renolfi prepared the manuscript; Eleonora Gambaro performed statistical analysis; Eleonora Gambaro, Carla Gramaglia, and Patrizia Zeppegno revised it critically for important intellectual content.

## ETHICS APPROVAL AND CONSENT TO PARTICIPATE

Approval for the research and informed consent are not necessary for this type of work.

### Peer Review

The peer review history for this article is available at https://publons.com/publon/10.1002/brb3.1803.

## Data Availability

The datasets used and/or analyzed during the current study are available from the corresponding author on reasonable request.
